# Chromosome painting and its applications in cultivated and wild rice

**DOI:** 10.1186/s12870-018-1325-2

**Published:** 2018-06-07

**Authors:** Lili Hou, Meng Xu, Tao Zhang, Zhihao Xu, Weiyun Wang, Jianxiang Zhang, Meimei Yu, Wen Ji, Cenwen Zhu, Zhiyun Gong, Minghong Gu, Jiming Jiang, Hengxiu Yu

**Affiliations:** 1 Key Laboratory of Plant Functional Genomics of Ministry of Education/Jiangsu Key Laboratory of Crop Genetics and Physiology/Co-Innovation Center for Modern Production Technology of Grain Crops, Yangzhou University, Yangzhou, 225009 China; 2 Department of Horticulture, University of Wisconsin-Madison|, Madison, WI 53706 USA

**Keywords:** *Oryza sativa*, *Oryza eichingeri*, FISH, Chromosome painting, Oligonucleotide, Chromosome variation

## Abstract

**Background:**

The chromosome-specific probe is a fundamental tool of chromosome painting and has been commonly applied in mammalian species. The technology, however, has not been widely applied in plants due to a lack of methodologies for probe development. Identification and labeling of a large number of oligonucleotides (oligos) specific to a single chromosome offers us an opportunity to establish chromosome-specific probes in plants. However, never before has whole chromosome painting been performed in rice.

**Results:**

We developed a pooled chromosome 9-specific probe in rice, which contains 25,000 oligos based on the genome sequence of a *japonica* rice (*Oryza sativa* L*.*, AA, 2n = 2× = 24). Chromosome 9 was easily identified in both *japonica* and *indica* rice using this chromosome 9-painting probe. The probe was also successfully used to identify and characterize chromosome 9 in additional lines of *O. sativa*, a translocation line, two new aneuploids associated with chromosome 9 and a wild rice (*Oryza eichingeri* A. Peter, CC, 2n = 2× = 24).

**Conclusion:**

The study reveals that a pool of oligos specific to a chromosome is a useful tool for chromosome painting in rice.

**Electronic supplementary material:**

The online version of this article (10.1186/s12870-018-1325-2) contains supplementary material, which is available to authorized users.

## Background

Chromosome painting (CP) is a technique of fluorescence in situ hybridization (FISH), which offers a powerful method for detection of specific chromosome regions or entire chromosomes based on chromosome-specific probes [1, 2]. In the past few decades, this technique has been primarily used to study human and animal chromosomes for diagnosing chromosome abnormalities, investigating chromosome rearrangements during evolution, and constructing ancestral karyotypes [3–7]. It has also been applied to molecular cytotaxonomy [8–11]. Chromosome painting probes in these cases were derived from flow-sorted or micro-dissected chromosomes followed by degenerate oligonucleotide-primed PCR amplification [12–14].

For most plant genomes, DNA probes prepared from flow-sorted or micro-dissected plant chromosomes failed to yield satisfactory results due to the presence of vast amounts of repetitive DNA that cannot be efficiently eliminated or blocked in FISH experiments [15–18]. To overcome such difficulties, a new FISH technique using large-insert DNA clones [bacterial artificial chromosomes (BACs) or yeast artificial chromosomes (YACs)] was developed for some plant species with small genomes. Fransz et al. used several YACs to paint specific regions of *Arabidopsis thaliana* L. chromosome 4 [19]. Contiguous BACs were also grouped as probes to paint an individual chromosome in *A. thaliana* [20] and *Brachypodium distachyon* L. [15]. This chromosome painting technique is a powerful tool to study genome duplication, chromosomal rearrangement, and evolution in species associated with *A. thaliana* and *B. distachyon* [21–25]. However, this technique requires ordered BAC contigs that cover the entire genome of a plant species. More importantly, it relies on the fact that both *A. thaliana* and *B. distachyon* have relatively small genomes. Most of the selected BACs contain almost exclusively single- or low-copy sequences.

Another successful chromosome painting method is based on PCR amplification to pool a large number of single-copy sequences. Lou et al. applied this method and obtained seven gene pool probes with sizes above 2 kb and an interval less than 300 kb to successfully paint each pachytene chromosome pair in *Cucumis sativus* L. [26]. A cucumber karyotype was also constructed based on these chromosome-specific probes and a comparative chromosome map of a region on chromosome 4 was constructed for both cucumber and melon. Unfortunately, amplification of a large number of PCR products that cover an entire chromosome is especially difficult and time consuming for plant species with large and complex genomes.

Sequence availability of the target DNA and massive parallel synthesis techniques make it possible to produce complex oligonucleotide (oligo) library that consist of thousands of independent oligos. Moreover, this kind of probes has been used successfully as FISH probes in mammalian and *Drosophila* species [27–29]. Han et al. first applied this technique in plants [30]. Oligo library specific to a single chromosome of cucumber was identified using a bioinformatics pipeline and then oligos were massively synthesized de novo in parallel. The synthesized oligos were amplified and labeled with biotin or digoxigenin, and were used as probes in FISH. They developed three different probes with each containing 23,000–27,000 oligos. These probes spanned 8.3–17 Mb of DNA on targeted cucumber chromosomes and had a density of 1.5–3.2 oligos per kilo base pairs. These oligo probes can be used to track homeologous chromosome pairing in early meiotic stages and examine the chromosome pairing behavior between homeologous chromosomes of cucumber. Divergence between *Cucumis melo* L. and an African *Cucumis* species were identified by chromosome painting using an oligo library and combining rDNA distribution patterns [31]. This technique was also used successfully to study the sex chromosomes in *Populus tomentosa* Carr. and *Populus deltoids* March [32]. Braz, et al. developed a bar code containing 54,672 oligos to label chromosomes from both diploid and polyploid potato species. This probe can also identify the homeologous chromosomes among distantly related species of *Solanum* L., including tomato and eggplant [33].

The genus *Oryza* L. consists of more than 20 species, including about 20 wild *Oryza* species and two cultivated species. The genomes of the wild *Oryza* species can be classified into 10 distinct groups (A, B, BC, C, CD, D, E, F, G, HJ and HK). Except *O*. *brachyantha* A. Chev. & Roehr. (F genome), all species in the genus *Oryza* are grouped into four main species complexes: *sativa*, *officinalis*, *ridleyi* and *meyeriana*, based on classical taxonomy, isozyme, RFLP and gene sequences. Nine species with B, BC, C, CD and E genomes were grouped to the *officinalis* complex [34–36]. The two cultivated species, *O. sativa* L. and *O. glaberrima* Steud. with 24 chromosomes are grouped in A genome. Two sub species, *japonica* and *indica* were identified as *O. sativa* based on their morphology and growth habitats [35, 37, 38]. They were likely domesticated from a specific wild rice population of *O. rufipogon* W. Griffith (A genome) [39, 40]. *Oryza officinalis* Wall. ex Watt and *O. eichingeri* A. Peter are diploid species with C genomes (2n = 2× = 24). *Oryza officinalis* is considered to be the basic genome of the *officinalis* complex and is considered to be similar to the A genome of *O. sativa* [35, 41]. The wild rice with C genomes is an important resource of valuable traits for the improvement of cultivated rice, such as disease and insect resistances [42–45]. But there is less research on *O. eichingeri*. *Oryza sativa* is one of the most important crops and an excellent model plant for monocotyledonous plants because it possesses a small genome that has also been completely sequenced. Despite the fact that the genomic sequence of a *japonica* variety, Nipponbare, has been available for a long time [46], no whole-chromosome painting experiment has been performed in any rice species. To study the possibility of application of oligo probes in *O. sativa* and examine the differences between *O. sativa* and *O. eichingeri,* we developed a library containing 25,000 oligos for identification of chromosome 9 in rice. This probe generated bright and chromosome-specific FISH signals in *O. sativa*. We used it to confirm a translocation between chromosome 9 and 11, as well as identify two new aneuploids associated with chromosome 9. This chromosome 9-specific oligo library could also be useful to trace chromosome 9 of *O. eichingeri*.

## Results

### Establishment of a chromosome-specific oligonucleotide set

To develop the chromosome 9-specific painting probe, we identified all non-overlapping oligos that are unique to chromosome 9 of Nipponbare using the bioinformatics pipeline developed by Han et al. with some modification [30]. Oligos with homology to repetitive DNA sequences or to sequences located on other chromosomes were eliminated. The oligo set containing 25,000 oligos was selected basing on the density of two oligos per kilobase, covering the entire length of chromosome 9 of Nipponbare. Sequence data of the oligo library can be found in the Additional file 1.

### Sensitivity and reliability of oligo probe for a *japonica* rice

**Fig. 1:**
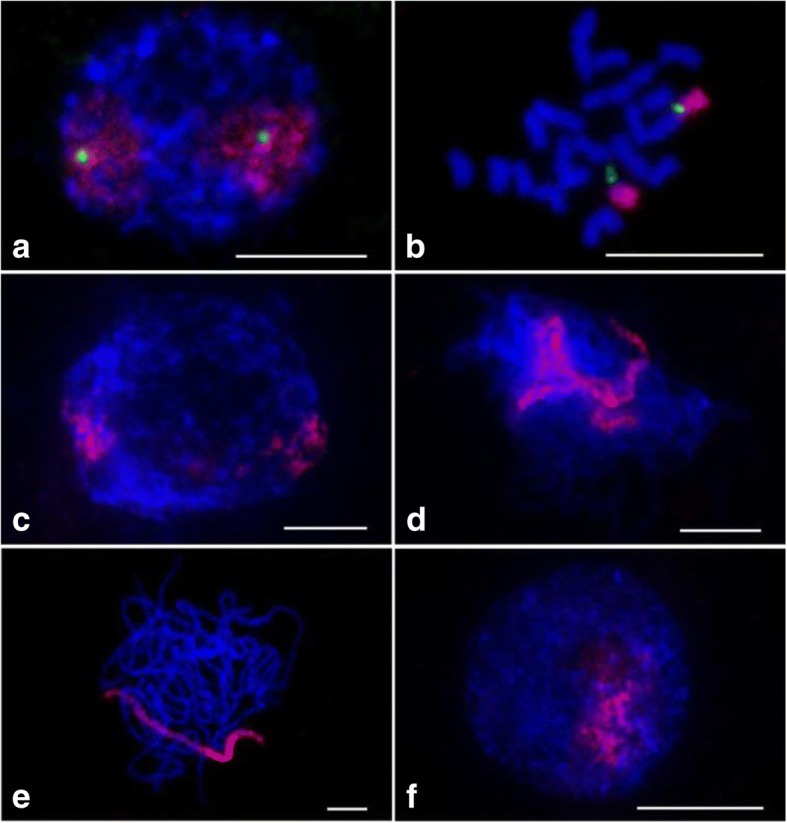
FISH using the chromosome 9-specific oligo library and 45S rDNA probes on chromosomes of Nipponbare. The chromosome 9-specific (red) and 45S rDNA probes (green) fluorescing in chromosomes in the (**a**) interphase, (**b**) prometaphase, (**c**) premeiotic interphase, (**d**) zygotene, (**e**) pachytene and (**f**) microspore. Chromosomes were stained with DAPI. Bars, 5 μm

For testing the sensitivity and reliability of the oligo probe for chromosome 9, a digoxigenin–labeled oligo set and a biotin-labeled 45S ribosomal RNA gene(45S rDNA)probe were hybridized to interphase and prometaphase chromosomes which were prepared from root tips of Nipponbare plants. The 45S rDNA signal is typically located at the telomeric region on chromosome 9 in *japonica* rice [47]. Both probes produced bright FISH signals on interphase nuclei and prometaphase chromosomes (Fig. 1a and b). Signal of the chromosome 9-specific probe occupied two separate domains on interphase nuclei (Fig. 1a). As expected, the FISH signal from the oligo probe (red) nearly uniformly covered the entirety of chromosome 9 without signal gaps. No cross-hybridization signal was detected on any other chromosomes. The 45S rDNA signals (green) were detected at the ends of the short arms of chromosome 9. To demonstrate the convenience of the oligos for chromosome painting in meiosis, the chromosome 9 specific probe was also hybridized to meiotic chromosomes of Nipponbare (Fig. 1c, d, e and f). During premeiotic interphase, two painted chromosome 9 were detected. They were separated from each other (Fig. 1c). However, during the late zygotene stage, the two painted chromosome 9 aligned very well (Fig. 1d). Fully synapsed chromosome 9 was observed at the pachytene stage (Fig. 1e). However, only one chromosome harbored the FISH signal in the microspore, indicating that the chromosome number was halved in the nucleus after meiosis (Fig. 1f). In addition, the intensity of the FISH signal on the zygotene and pachytene chromosomes was not as strong as that on mitotic prometaphase chromosomes. These results confirm that chromosome 9-specific probe is sensitive and reliable for identifying both mitotic and meiotic chromosomes of rice in CP experiments.

### Delimitation of chromosome 9 of an *indica* rice and identification of chromosome translocation using a chromosome 9-specific oligo probe

**Fig. 2:**
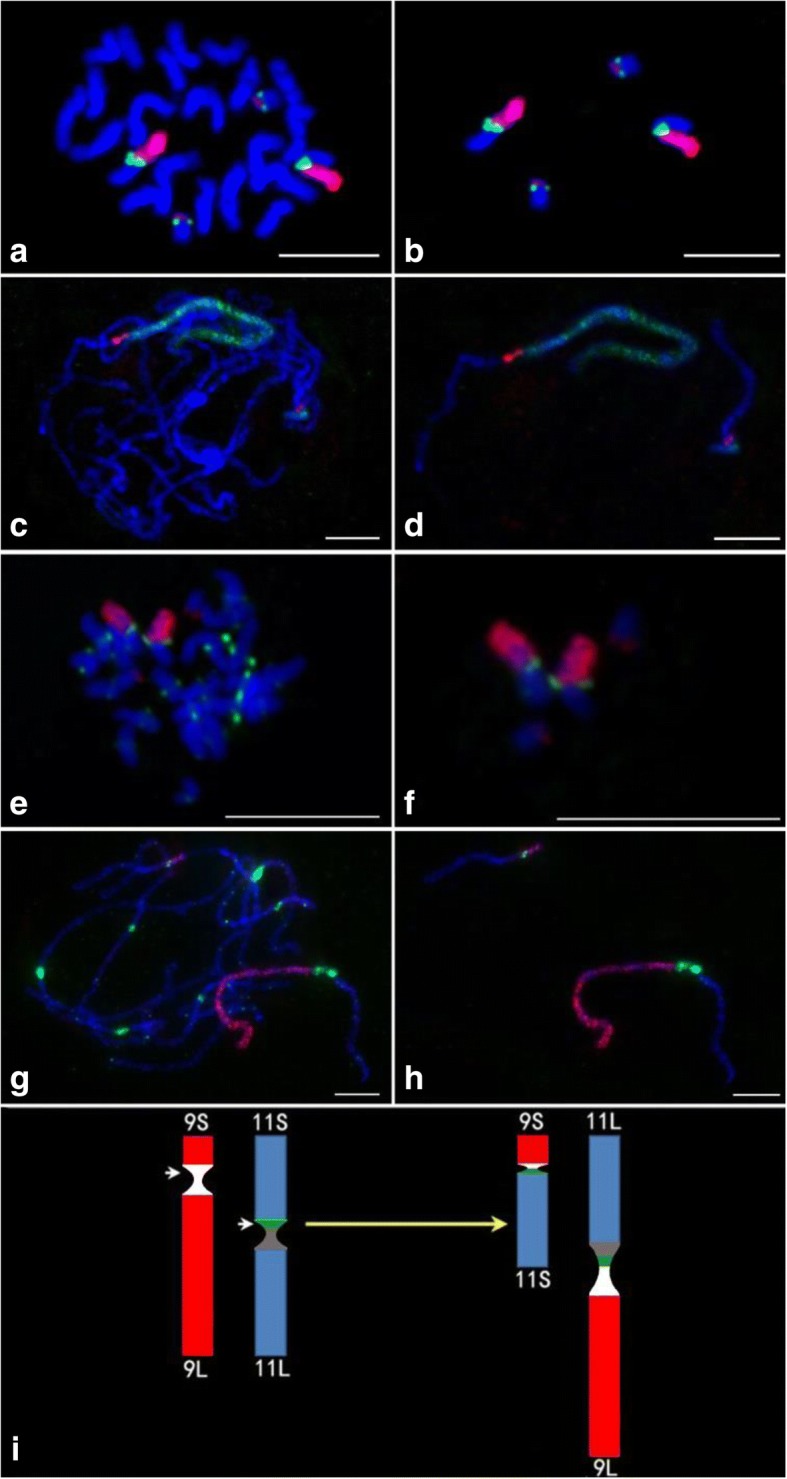
Identification of a Chr9/11 translocation in an *indica* rice, Zhongxian 3037 by FISH. **a** The chromosome 9-specific (red) and 5S rDNA (green) probes on mitotic prometaphase chromosomes. **c** The chromosome 9-specific (green) and 5S rDNA (red) probes on chromosomes at the pachytene stage. The chromosome 9-specific (red) and CentO (green) probes fluorescing in chromosomes in the (**e**) mitotic prometaphase and (**g**) pachytene. **b** and **f** Two pairs of translocation chromosomes were digitally separated from the rest of the chromosomes shown in panels (**a**) and (**e**), respectively. **d** and **h** Two bivalents of the translocation chromosomes were digitally separated from the rest of the chromosomes shown in panels (**c**) and (**g**), respectively. Chromosomes were stained with DAPI. **i** The idiograms of chromosomes 9 and chromosome 11 of Zhongxian 3037 (left) and Chr9S·11S and Chr11L·9 L of the translocation line (right). The white region is the centromere of chromosome 9. The green region is 5S rDNA and the gray region is the centromere of chromosome 11. White arrows indicate the breakpoints. Bars, 5 μm

To test if the chromosome 9-specific probe selected based on a *japonica* rice would work for *indica* rice and confirm the chromosome rearrangement in a translocation line derived from an *indica* rice Zhongxian 3037 [48], the chromosome 9-specific oligo library and 5S ribosomal RNA genes(5S rDNA)were used as probes in the FISH experiment. The 5S rDNA locates on the short arm of rice chromosome 11 close to the centromere [49]. In normal mitotic prometaphase cells of Zhongxian 3037, 5S rDNA signals can be detected on two chromosomes. However, during mitotic prometaphase, the signals of both the digoxigenin–labeled chromosomes 9- specific (red) and biotin-labeled 5S rDNA probes (green) were observed closely adjacent to each other on four chromosomes in the translocation line (Fig. 2a and b). Furthermore, the translocation chromosomes with the long arms of chromosome 11 had brighter signals of 5S rDNA. These results indicate that a reciprocal translocation had happened between chromosomes 9 and 11 and the translocation breakpoint was likely located within the 5S rDNA array on chromosome 11. During the pachytene stage, both probes produced bright FISH signals on two bivalents of this mutant (Fig. 2c and d). Chromosomes 9 (green) and 5S rDNA (red) signals were closely adjacent to each other on both bivalents, which also confirmed that the translocation occurred between chromosome 9 and 11. Thus chromosome 9-specific oligo probe selected based on a *japonica* rice also performed well in an *indica* rice.

To further characterize the translocation chromosome, the 155-bp CentO repeat, which is specific to centromeres of rice chromosomes [50] and the chromosome 9-specific probe were used for FISH. Chromosome 9 is an acrocentric chromosome and the content of CentO in chromosome 11 is about two times that of chromosome 9 in Zhongxian 3037 [51]. In the translocation stock, a strong CentO signal (green) was detected on the longer translocation chromosomes (Fig. 2e and f). CentO signal was not consistently detected on the shorter translocation chromosomes during mitotic prometaphase (Fig. 2e and f). However, clear CentO signals were detected on both the long and short translocation chromosomes at the pachytene stage (Fig. 2g and h). Interestingly, two separate flourescing blocks of CentO signals were observed on the longer translocation bivalent. The CentO signal was weaker on the shorter translocation bivalent, indicating that the shorter translocation chromosomes harbored the smaller parts of the centromeres of chromosome 9. The longer translocation chromosomes were designated as 9 L.11 L because they harbored the long arms of chromosome 9 (red) and chromosome 11 (blue). Whereas the shorter translocation chromosomes, designated as 9S.11S had the short arms of chromosome 9 and chromosome 11. Taken together, the breakpoint in chromosome 9 occurred in the CentO array, which resulted in two CentO arrays; the large array was received by 11 L.9 L and the small one was received by 11S·9S (Fig. 2i). The breakpoint in chromosome 11 occurred in the middle of the 5S rDNA locus, which resulted in 5S rDNA signals on both the 11 L.9 L and 11S.9S chromosomes (Fig. 2i).

### Characterization of rice aneuploids associated with chromosome 9

**Fig. 3:**
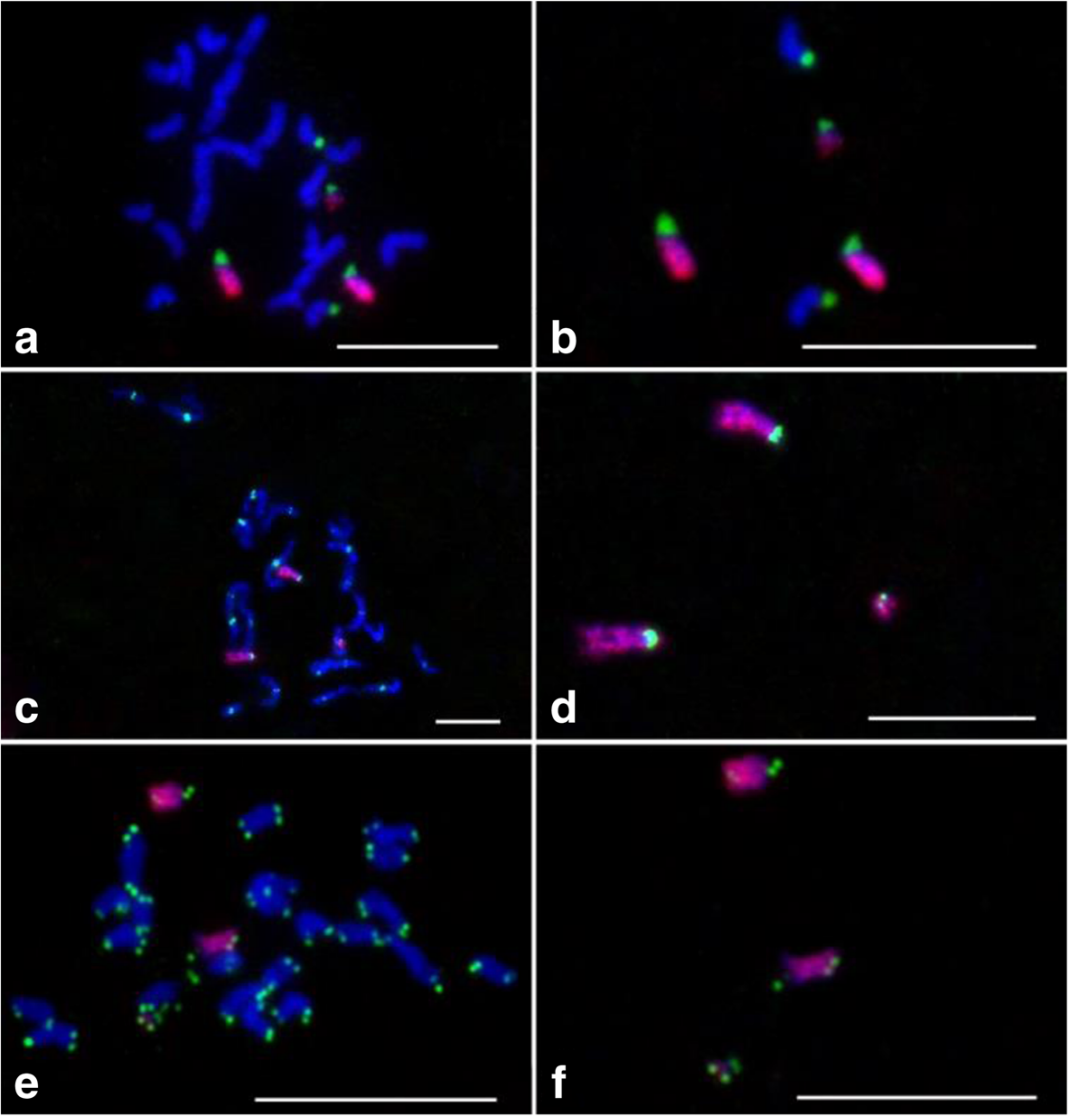
Localization of chromosome 9-specific oligo library, CentO, pAtT4 and 45S rDNA probes on the chromosomes of aneuploid YN6077. **a** The chromosome 9-specific (red) and 45S rDNA (green) probes on mitotic prometaphase chromosomes. **b** Five chromosomes with 45S rDNA signals were digitally separated from the rest of the chromosomes shown in panel (**a**). **c** The chromosome 9-specific (red) and CentO (green) probes on mitotic prometaphase chromosomes. **e** The chromosome 9-specific (red) and pAtT4 (green) probes on mitotic prometaphase chromosomes. **d** and **f** Three chromosomes with chromosome 9-specific signals were digitally separated from the rest of the chromosomes shown in panels (**c**) and (**e**), respectively. Chromosomes were stained with DAPI. Bars, 5 μm

To identify and characterize aneuploids associated with chromosome 9, we selected putative aneuploid plants with different phenotypes compared to the trisomic 9 and Zhongxian 3037 from progenies of a rice trisomic 9 (2n = 25, with three copies of chromosome 9). To investigate the potential chromosomal mutation in these plants, 45S rDNA, CentO and the chromosome 9-specific oligo probes were used in FISH analyses. The 45S rDNA signal is typically located at telomeric regions on both chromosome 9 and 10 in Zhongxian 3037 [47]. The mutant line, YN6077 contained 25 chromosomes, including a pair of normal chromosome 9 and an additional short chromosome. This short chromosome bore a chromosome 9-specific signal (red), and the 45S rDNA signal (green) located at one end (Fig. 3a and b). The signal of CentO on this short chromosome was weaker than that on the normal chromosome 9 and the short chromosome also had two arms with different lengths (Fig. 3c and d). To further characterize this chromosome, a telomeric DNA probe, pAtT4 and chromosome 9-specific oligo probe were used for FISH (Fig. 3e and f). The telomeric probe signals (green) were located on the two ends of this short chromosome. These results suggested that both arms of this short chromosome were derived from chromosome 9 and one arm was the short arm of chromosome 9. The exact components of the short chromosome require further verification.

**Fig. 4:**
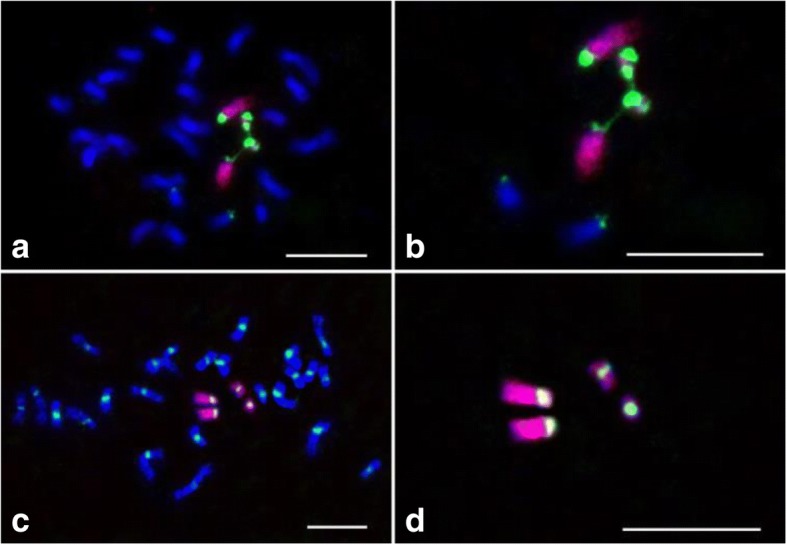
FISH visualization of the localization of chromosome 9-specific oligo library, CentO, and 45S rDNA probes on the chromosomes of aneuploid YN6089. **a** The chromosome 9-specific (red) and 45S rDNA (green) probes on mitotic prometaphase chromosomes. **b** Six chromosomes with 45S rDNA signals were digitally separated from the rest of the chromosomes shown in panel (**a**). **c** The chromosome 9-specific (red) and CentO (green) probes on mitotic prometaphase chromosomes. **d** Four chromosomes with chromosome 9-specific signals were digitally separated from the rest of the chromosomes shown in panel (**c**). Chromosomes were stained with DAPI. Bars, 5 μm

We detected 28 chromosomes in mutant YN6098 (Fig. 4), including two normal chromosome 9 and two additional small chromosomes with chromosome 9-specific signals. These two small chromosomes contained 45S rDNA signals at both ends (Fig. 4a and b). The CentO signals were located in the middle of these two chromosomes (Fig. 4c and d). These results showed that the small chromosomes are isochromosomes derived from the short arm of chromosome 9.

### Cross-species chromosome painting using the oligo probe

**Fig. 5:**
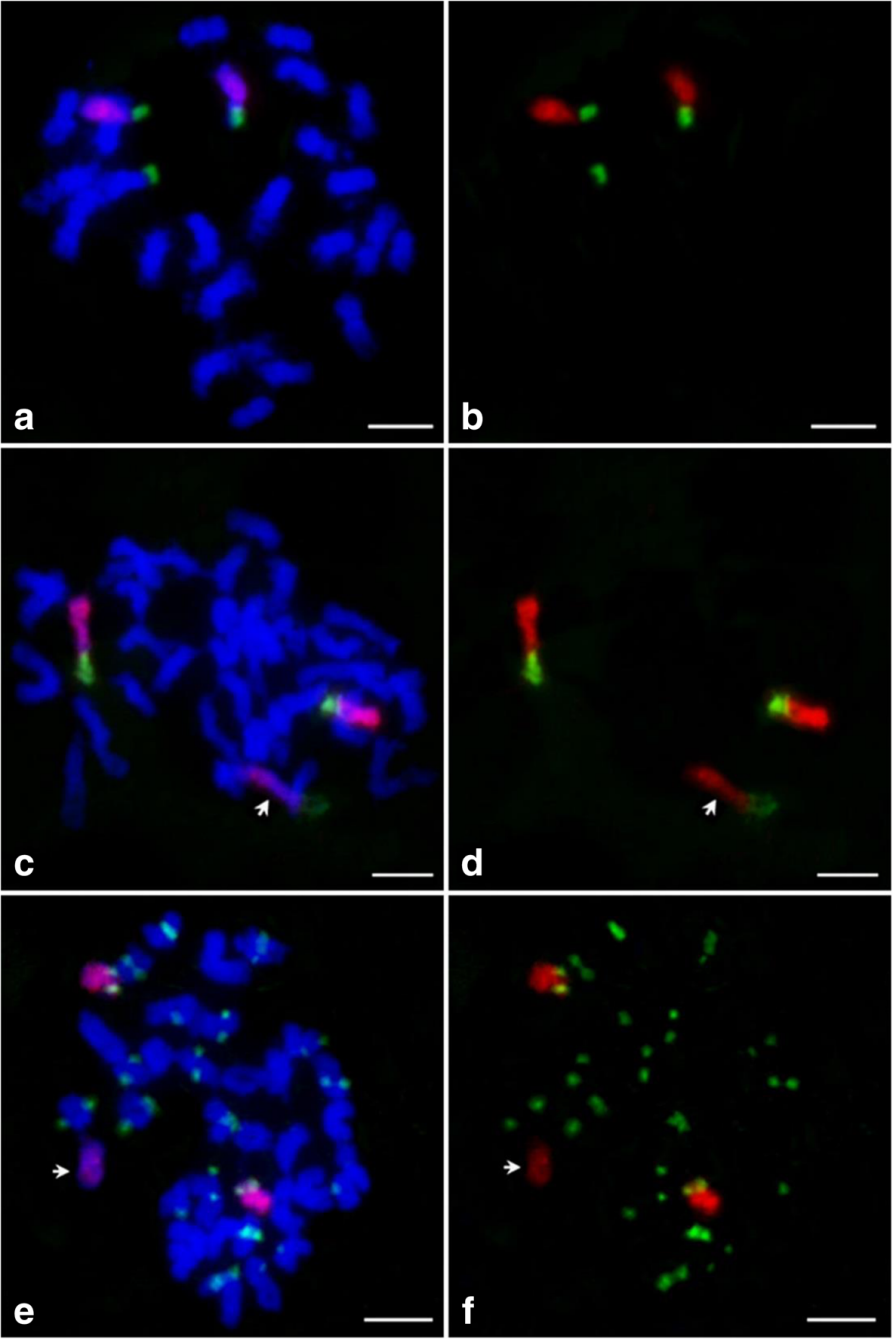
Localization of the chromosome 9-specific oligo library, 45S rDNA and CentO probes on the chromosomes of *O. eichingeri* and a triploid (AAC). The chromosome 9-specific (red) and 45S rDNA probes (green) fluorescing in prometaphase chromosomes of the (**a**) *O. eichingeri* (CC, 2n = 2× = 24) and (**c**) the triploid (AAC, 2n = 3× = 36) derived from *O. eichingeri* and 02428. **b** and **d** The chromosome 9-specific and 45S rDNA signals were digitally separated from the rest of the chromosomes in panels (**a**) and (**c**), respectively. **e** The chromosome 9-specific (red) and CentO (green) probes on chromosomes of the triploid. **f** The chromosome 9-specific and CentO signals were digitally separated from the rest of the chromosomes in (**e**). White arrows indicate the chromosome 9 from *O. eichingeri*. Chromosomes were stained with DAPI. Bars, 5 μm

To test the potential application of the chromosome 9 specific oligo probe on other genomes in the genus *Oryza*, we conducted a FISH assay using the chromosome 9-specific oligo and 45S rDNA probes on the mitotic prometaphase chromosomes of *O. eichingeri* (CC, 2n = 2× = 24). Distinct FISH signals of chromosome 9 and 45S rDNA were detected on two and three chromosomes in each cell (Fig. 5a and b). For comparing chromosome 9 between *O. eichingeri* and *O. sativa,* the chromosome 9-specific oligo and 45S rDNA probes were utilized in a FISH experiment on mitotic prometaphase chromosomes which were prepared from the root tips of a triploid line (AAC, 2n = 3× = 36) derived from the *O. eichingeri* and a *japonica* rice, 02428. Distinct FISH signals were detected on three chromosomes in each cell (Fig. 5c and d). However, the FISH signals of the chromosome 9-specific oligo (red) and 45S rDNA (green) probes on one chromosome were weaker. To examine further the origins of the three chromosomes with chromosome 9 signal, the chromosome 9 specific oligo and CentO probes were used in FISH experiments (Fig. 5e and f). The chromosome with weaker chromosome 9 signal harbored no CentO signal and the CentO signals were observed on the two chromosomes with brighter chromosome 9 signals. These results indicated that the chromosome with weaker chromosome 9-specific signal is derived from *O. eichingeri*, as no CentO, a centromeric satellite on chromosome 9 of *O. eichingeri* [52]. And this chromosome 9 specific oligo probe can be used to track chromosome 9 of *O. eichingeri*.

## Discussion

A chromosome-specific oligo probe offers a powerful way to identify a specific chromosome both in mitosis and meiosis in a *Cucumis* species [30]. Our experiment also demonstrated that the oligo probe based on the sequence of a *japonica* rice, can be used to identify chromosome 9 in both *japonica* and *indica* rice. This probe is also a useful tool to characterize the variations of related chromosomes. In this study, a chromosome 9-specific oligo set was used to characterize the variations of chromosome 9 in different mutants. This system appears to be more efficient and reliable than the traditional marker-based methods to detect chromosome variation. For example, it would be difficult to characterize the double isochromosome 9S in line YN6089 (Fig. 4) using the traditional marker-based methods because the presence of six copies of 9S in this line would complicate the interpretation of the marker-based data.

The two arms of the additional short chromosome had the signals of chromosome 9 in the mutant line, YN6077. However, the 45S rDNA signal was detected only at one end of this chromosome. The arm without 45S rDNA in this chromosome is very short. We surmised a possible explanation for the occurrence of the short arm based on the fact that this mutant was derived from a trisomic 9. It may be a part of the long or short arm of chromosome 9. Moreover, a rearrangement involving the centromere may have also occurred in this short chromosome. To determine the components of this chromosome, chromosome 9 arm-specific or fragment-specific oligo probes may be used in future studies. Of course, as the technology on array-based comparative genomic hybridization continues to improve, we can continue to improve our ability to more rapidly and accurately identify chromosomal copy number variation in rice and other plant species [53, 54].

Oligo-based chromosome painting can potentially be used to detect chromosomes or chromosomal segments in germplasm stocks. This approach has been used successfully to paint homeologous chromosomes from several diploid and polyploidy *Cucumis* species that diverged from cucumber up to 12 million years ago. Pairing of a cucumber chromosome with a specific homeologous chromosome from a wild species can be monitored in the hybrid [30, 31]. In the present study, chromosome 9-specific probe based on a *japonica* rice also exhibited similar signals for chromosome 9 in *O. eichingeri* (C genome). This indicates that sequence similarity exists in chromosome 9 between *O. sativa* and *O. eichingeri*. It is consistent with the result that *O. sativa* and *O. officinalis* share a high degree of synteny of RFLP markers [55]. However, the FISH signals of both oligo probes and 45S rDNA on chromosome 9 in *O. eichingeri* were much weaker than those in *O. sativa*, indicating that some differentiation has been occurred between *O. eichingeri* and *O. sativa* [35]. Thus, this chromosome specific oligo probe can be used to track homeologous chromosome pairing in the hybrids derived from *O. sativa* and *O. eichingeri*. However, whether this probe would be able to efficiently distinguish chromosome 9 among other rice genomes and monitor the homeologous chromosome pairing in their heterogeneous hybrids remains elusive.

## Conclusions

The chromosome-specific probe is a fundamental tool of chromosome painting. We developed a pooled chromosome 9-specific probe in rice, which contains 25,000 oligos based on the genome sequence of a *japonica* rice (*O. sativa*, AA, 2n = 2× = 24). Chromosome 9 was easily identified in both *japonica* and *indica* rice using this chromosome 9-painting probe. The probe was also successfully used to identify and characterize chromosome 9 in additional lines of *O. sativa*, a translocation line, two new aneuploids associated with chromosome 9 and a wild rice (*O. eichingeri*, CC, 2n = 2× = 24). The study reveals that a pool of oligos specific to a chromosome is a useful tool for chromosome painting in rice.

## Methods

### Plant materials

A total of six kinds of rice were used in this study: one cultivar of *japonica* rice, Nipponbare (*O. sativa*, AA, 2n = 2× = 24); two aneuploids derived from the progeny of a trisomic 9 of the *indica* rice Zhongxian 3037 (*O. sativa*); a translocation line with reciprocal translocation between chromosomes 9 and 11 in Zhongxian 3037 [48]; a wild rice, *O. eichingeri* (CC, 2n = 2× = 24) and a triploid (AAC, 2n = 3× = 36) derived from *O. eichingeri* (CC, 2n = 2× = 24) and a *japonica* rice 02428 [45]. All materials were planted in the trial fields of Yangzhou University (Yangzhou, Jiangsu Province, China).

Root tips were harvested from rice plants and pretreated in 0.002 M 8-hydroxyquinoline at 20 °C for 2 h, then fixed in methanol-acetic acid (3:1) and stored at − 20 °C until use. Squashes of root tips were prepared according to Yu et al. [56]. Young panicles of rice plants were harvested and fixed in Carnoy’s solution (ethanol: glacial acetic acid, 3: 1) and stored at − 20 °C. Squashes of panicles were prepared in acetic-carmine solution according to Cheng et al. [57].

### Bioinformatics pipeline for oligo selection

We used the Chorus software (https://github.com/forrestzhang/Chorus) for selecting chromosome 9-specific oligos [30]. In general, we used the rice genome (TIGR7, http://rice.plantbiology.msu.edu/) as reference [58]. All repeat sequences of chromosome 9 were filtered by applying RepeatMasker (http://www.repeatmasker.org). We divided repeat filtered chromosome 9 sequences into 45 nt with a step size of 5 nt. Then we mapped all the oligo sequences to the whole genome of rice and filtered oligos which can map to two or more loci with 75% homology. Next, we calculated the melting temperature (Tm) and hairpin Tm of each oligo. Oligos with dTm > 10 (dTm = Tm-hairpin Tm) were kept to build a probe set.

### Probe preparation and FISH

The oligo library was synthesized by MYcroarray (Ann Arbor, MI, USA). Probe preparation from the synthesized oligo library was conducted as described by Han et al. [30]. FISH analysis was performed as described by Han et al. [30]. The biotin- or digoxigenin-labeled single-stranded oligos prepared from the library were directly used as FISH probes. CentO which contains a 155-bp satellite repeat of a rice centromere [50], a pTa794 clone containing the coding sequence for the 5S rDNA of wheat [59], 45S rDNA [47] and a pAtT4 clone containing *A. thaliana* telomeric DNA [60] were labeled with either digoxigenin-11-dUTP or biotin-dUTP (Roche) by standard nick translation and included in FISH probes [61].

Each probe was detected using a fluorescein isothiocyanate conjugated anti-biotin or anti-digoxigenin antibody (Vector Laboratories). The chromosomes were counterstained with 4′, 6′-diamidino-phenylindole (DAPI) in Vectashield antifading solution (Vector laboratories). Chromosomes and FISH signals were observed under an Olympus BX61 fluorescence microscope and images were captured with a DVC1412 CCD camera using IPLab software.

## Additional file

Additional file 1: The chromosome 9-specific oligo sequence data. (BED 1827 kb)

## Abbreviations

45S rDNA: 45S ribosomal RNA gene
5S rDNA: 5S ribosomal RNA gene
BAC: Bacterial artificial chromosome
CP: Chromosome painting
DAPI: 4′, 6′-diamidino-phenylindole
FISH: Fluorescence in situ hybridization
oligos: Oligonucleotides
Tm: Melting temperature
YAC: Yeast artificial chromosomes
